# Determining the ED90 of prophylactic phenylephrine and norepinephrine infusions for higher blood pressure target maintenance during cesarean delivery: a randomized sequential allocation trial

**DOI:** 10.3389/fphar.2026.1781654

**Published:** 2026-04-29

**Authors:** Yongqiang Shi, Rong Ma, Yueji Ma, Nan Xi, Yi Chen

**Affiliations:** Department of Anesthesiology and Perioperative Medicine, General Hospital of Ningxia Medical University, Yinchuan, China

**Keywords:** blood bressure management, cesarean delivery, norepinephrine, phenylephrine, sequential allocation

## Abstract

**Background:**

The international consensus on vasopressors for managing hypotension emphasizes maintaining maternal systolic blood pressure (SBP) during cesarean delivery greater than 90% of the baseline and not below 80%. This study was to determine the ED90 values of prophylactic norepinephrine and phenylephrine infusions under higher blood pressure management target of maintaining maternal SBP at greater than 90% of the baseline.

**Method:**

Sixty patients aged 18–45 years, who were scheduled to undergo cesarean delivery under spinal anesthesia, were randomly assigned to the norepinephrine group and the phenylephrine group. The first patients in each group were administered prophylactic infusion rates of 0.05 and 0.5 μg/kg/min, respectively. Effectiveness was determined based on whether the current vasopressor infusion dose could achieve the predefined target for maternal SBP management at greater than 90% of the baseline prior to fetal delivery. Subsequently, the vasopressor infusion rate was adjusted incrementally or decrementally by 0.01 and 0.1 μg/kg/min as needed. Additionally, maternal adverse events and neonatal outcomes were systematically recorded and analyzed.

**Results:**

Through isotonic regression analysis, the ED90 values for prophylactic norepinephrine and phenylephrine infusions under a higher blood pressure management target were estimated to be 0.140 μg/kg/min (95% CI: 0.133–0.147) and 1.280 μg/kg/min (95% CI: 1.180–1.380), respectively. There was no statistically significant difference in the incidence of adverse events or neonatal outcomes between groups.

**Conclusion:**

When the target was established to maintain the blood pressure of patients at greater than 90% of the baseline prior to delivery in patients undergoing cesarean delivery, the prophylactic infusion rates for norepinephrine and phenylephrine were determined to be 0.140 and 1.280 μg/kg/min, respectively.

**Clinical Trial Registration:**

Clinicaltrials.gov, NCT06158048.

## 1 Introduction

Maternal hypotension, the most prevalent complication during cesarean delivery, may lead to reduced perfusion in both the patient and the placenta, potentially compromising maternal comfort and inducing ischemic hypoxia in the fetus, which in severe cases can adversely impact the safety and long-term outcomes of both the patient and the neonate (Lyu et al., 2023; Mohta et al., 2019; Wei et al., 2020).

The international consensus on the use of vasopressors for the management of hypotension emphasizes maintaining intraoperative blood pressure during cesarean delivery at greater than 90% of the baseline while preventing it from falling below 80%, and it also recommends the prophylactic administration of phenylephrine at a variable rate (Kinsella et al., 2018). The American Society of Anesthesiologists (ASA) guidelines for obstetric anesthesia recommend phenylephrine as the first-line vasopressor for preventing and treating maternal hypotension during cesarean delivery (Practice Guidelines for Obstetric Anesthesia, 2016). However, due to its β-receptor agonist properties, norepinephrine is more effective than phenylephrine in maintaining cardiac output, making it a critical alternative vasopressor for managing maternal hypotension during cesarean delivery (Heesen et al., 2020; Belin et al., 2023).

Our recent trial involving preeclamptic patients with higher blood pressure target maintenance demonstrated that the ED90 values of prophylactic norepinephrine and phenylephrine infusions were 0.076 μg/kg/min and 0.900 μg/kg/min respectively (Ma et al., 2025). Given the endothelial dysfunction and excessive sympathetic tone observed in preeclamptic patients (Russell, 2020), and considering the current paucity of evidence, the corresponding ED90 values in normotensive patients are expected to be significantly higher. We conducted a randomized sequence allocation dose-finding study to determine the ED90 values of prophylactic norepinephrine and phenylephrine infusions for maintaining maternal SBP at greater than 90% of the baseline in normotensive patients undergoing cesarean delivery.

## 2 Materials and methods

### 2.1 Study design and population

This study received approval from the Ethics Committee of the General Hospital of Ningxia Medical University (No. KYLL-2023–0373-3) and was conducted from January to April 2025 in compliance with the principles of the Helsinki Declaration and CONSORT guidelines. The study was prospectively registered on ClinicalTrials.gov (No. NCT06158048) prior to the initiation of the trial, and all patients provided written informed consent before undergoing any intervention. Patients aged between 18 and 45 years who were scheduled to undergo cesarean delivery under spinal anesthesia were included in the study. Patients with a body mass index (BMI) of ≥40 kg/m^2^, non-full-term pregnancies, eclampsia, chronic hypertension, baseline blood pressure of ≥140 mmHg, fetal distress, or known fetal developmental anomalies were excluded from the study.

### 2.2 Monitoring and grouping

All patients and neonates received identical nursing care. Prior to spinal anesthesia and cesarean delivery, continuous monitoring of vital signs was initiated, encompassing electrocardiogram (ECG), non-invasive arterial blood pressure (NBP), and transcutaneous pulse oxygen saturation (SpO_2_). The baseline values were determined as the mean of three consecutive measurements. An intravenous cannula (18G) was inserted into the upper extremity to facilitate the administration of crystalloids and vasopressors. Based on randomization conducted using SPSS software, patients were randomly assigned to the norepinephrine group and the phenylephrine group.

### 2.3 Spinal anesthesia and study protocol

Prior to spinal anesthesia, an anesthesiologist who was not involved in intraoperative management disclosed an opaque envelope to allocate the patient to the designated group. Furthermore, this anesthesiologist prepared the vasopressor for each patient according to the sequence allocation method and determined the infusion rate. The vasopressor infusion syringes were labeled to conceal any information regarding the vasopressor or its dosage. Patients and the anesthesiologists who administered intraoperative management were blinded to the group assignments and the specific dosages of the vasopressors used. Spinal anesthesia was administered at the L3-4 interspace with the patient positioned laterally. A 25G needle was utilized to puncture the dura mater, and 12.5 mg of 0.5% bupivacaine was subsequently injected into the subarachnoid space. Post-anesthesia, the surgical table was adjusted to a 15° left lateral tilt. The sensory blockade was assessed by pain sensation, ensuring that the T6 dermatome represented the highest level of sensory blockade. An immediate crystalloid coload was administered at a rate of 8 ml/kg.

As per the findings of Hasanin et al. (2019) the recommended initial infusion dose of norepinephrine is 0.05 μg/kg/min, and the international consensus suggested a phenylephrine dosage of 25–50 μg/min (Kinsella et al., 2018). The first patients in each group were administered prophylactic infusion rates of 0.05 and 0.5 μg/kg/min, respectively. If the effectiveness had been achieved, the infusion rate for subsequent patients in the norepinephrine group and the phenylephrine group was decreased by 0.01 μg/kg/min and 0.1 μg/kg/min (i.e., one-fifth of the initial dosage), respectively. Conversely, the infusion rate for subsequent patients in both groups was increased by 0.01 μg/kg/min and 0.1 μg/kg/min, respectively. The dosage of vasopressors administered to each patient was determined based on the efficacy of the dose given to the preceding patient. The maximum allowable doses for the norepinephrine group and the phenylephrine group were determined to be 0.15 μg/kg/min and 1.5 μg/kg/min, respectively.

### 2.4 Maternal and neonatal outcomes

The primary outcome was the ED90 values of prophylactic norepinephrine and phenylephrine infusions for maintaining maternal SBP at greater than 90% of the baseline. The secondary outcomes included the incidence of maternal hypotension (SBP <80% of baseline), severe maternal hypotension (SBP <60% of baseline), bradycardia (heart rate <60 beats per minute), and hypertension (SBP >120% of baseline) prior to delivery. In cases of hypotension, patients were administered 6 μg of norepinephrine and 60 μg of phenylephrine, which were diluted to 6 μg/ml and 60 μg/ml with normal saline, respectively. If these interventions were ineffective, the same dosages were repeated. For bradycardia, 0.5 mg of atropine was administered when the heart rate decreased to <50 beats per minute. For hypertension, the vasopressor infusion was paused until SBP returned to <120% of baseline, after which the infusion was resumed. Other secondary outcomes included the incidence of nausea and vomiting, analysis of umbilical artery blood gas, and the neonatal Apgar score.

### 2.5 Sample size calculation

The sample size was determined based on previously reported literature, with a range of 20–40 cases and multiple reversals deemed sufficient for the sample size analysis of up-and-down sequence allocation. Therefore, we decided to include 30 patients in both the norepinephrine group and the phenylephrine group (Oron et al., 2022).

### 2.6 Statistical analysis

Statistical analysis was conducted using IBM SPSS Statistics 25.0. Continuous variables were assessed for normality using the Kolmogorov-Smirnov test. Group comparisons for continuous variables were performed using either the Student’s t-test (for normally distributed data) or the Mann-Whitney U test (for non-normally distributed data). Results for continuous variables are presented as mean ± standard deviation for normally distributed data and interquartile range (IQR) for non-normally distributed data. Chi-square tests were employed to compare categorical variables, with results presented as percentages. Multivariate logistic regressions were employed to evaluate safety and comparability regarding secondary outcomes of maternal bradycardia and hypertension between the two groups. The ED90 values of prophylactic norepinephrine and phenylephrine infusions for maintaining maternal SBP at greater than 90% of the baseline were analyzed using isotonic regression analysis. A p-value of less than 0.05 was considered indicative of statistical significance.

## 3 Results

**FIGURE 1:**
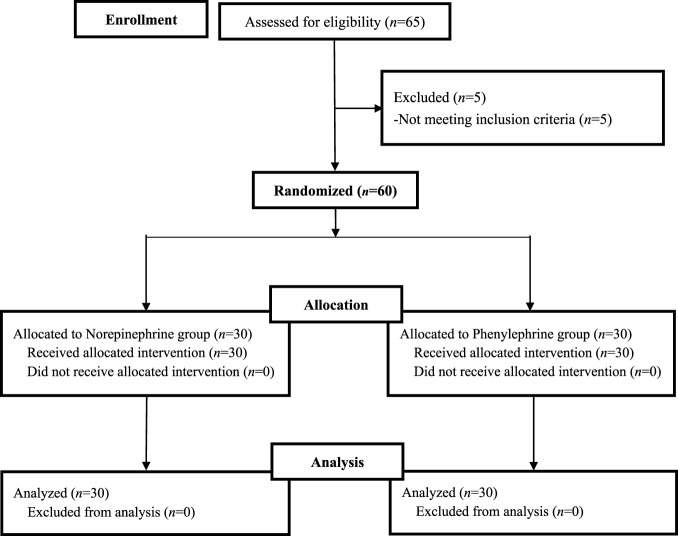
The inclusion flowchart.

The inclusion flowchart for the two groups of patients is illustrated in Figure 1, while their baseline and demographic characteristics are summarized in Table 1. No statistically significant differences were observed between the two groups.

**TABLE 1:** Maternal characteristics.

Characteristics	Norepinephrine group (n = 30)	Phenylephrine group (n = 30)	*P* value
Age, years	31.13 ± 3.89	31.73 ± 3.71	0.544
Body mass index, kg/m^2^	27.97 ± 3.37	29.27 ± 3.13	0.127
Gestational age, weeks	39 [38, 39]	39 [38, 39]	0.918
Gravidity	2 [1, 3]	2 [2, 3]	0.368
Parity	0 [0, 1]	1 [0, 1]	0.089
Maternal baseline characteristics			
Systolic blood pressure, mmHg	120.30 ± 9.21	118.17 ± 9.30	0.376
Heart rate, bpm	98.27 ± 11.17	93.40 ± 13.51	0.134
Sensory block	6 [6, 6]	6 [6, 6]	0.913
Anesthesia to fetal delivery interval, min	14.23 ± 2.66	15.34 ± 3.85	0.201
Incision to fetal delivery interval, min	3.33 ± 1.42	3.80 ± 1.61	0.238

Values are mean ± SD, or median [IQR].

**FIGURE 2:**
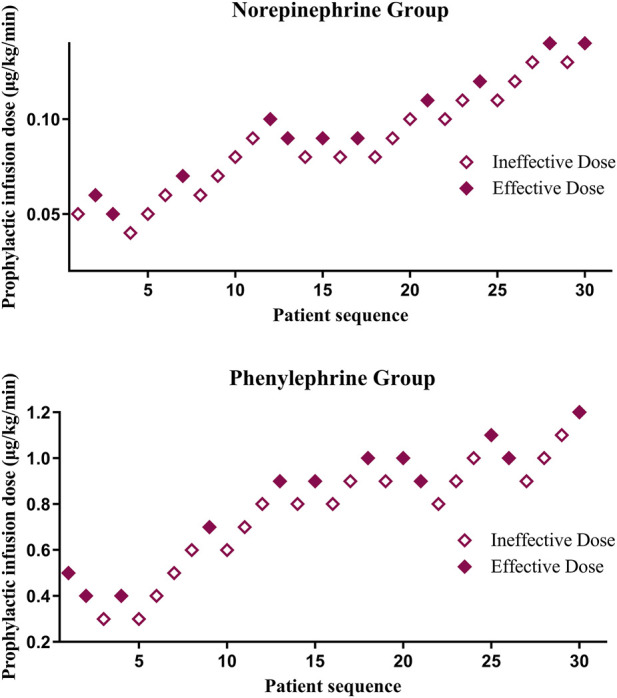
The sequences for the norepinephrine and phenylephrine groups.

The sequences for the two groups of patients are presented in Figure 2. The ED90 values for prophylactic norepinephrine and phenylephrine infusions under higher blood pressure management target were 0.140 μg/kg/min (95% CI, 0.133–0.147) and 1.280 μg/kg/min (95% CI, 1.180–1.380), respectively.

As presented in Table 2, there was no statistically significant difference in adverse events or neonatal outcomes between the two groups. Multivariate regression analysis indicated that a range of confounding variables had no influence on the comparability of maternal bradycardia and hypertension between the groups. Regarding maternal bradycardia, the confounding variables we included were intervention group (OR = 1.237; 95% CI: 0.030 to 51.208, *P* = 0.911), vasopressor dose (OR = 0.043; 95% CI: 0.005 to 37.762, *P* = 0.712), baseline HR (OR = 1.015; 95% CI: 0.945 to 1.090, *P* = 0.679), BMI (OR = 0.846; 95% CI: 0.638 to 1.121, *P* = 0.245), and sensory block (OR = 1.738; 95% CI: 0.466 to 6.480, *P* = 0.410). Regarding maternal hypertension, the confounding variables we included were treatment group (OR = 0.306; 95% CI, 0.004 to 21.790, *P* = 0.587), vasopressor dose (0.200; 95% CI, 0.001 to 59.159, *P* = 0.579), baseline SBP (OR = 1.088; 95% CI, 0.990 to 1.195, *P* = 0.081), age (OR = 1.175; 95% CI, 0.913 to 1.511, *P* = 0.210), parity (OR = 1.524; 95% CI, 0.397 to 5.854, *P* = 0.539).

**TABLE 2:** Maternal and neonatal outcomes.

Outcomes	Norepinephrine group (n = 30)	Phenylephrine group (n = 30)	*P* value
Maternal outcomes			
Post-spinal anesthesia hypotension, n (%)	19 (63.3)	17 (56.7)	0.972
Severe post-spinal anesthesia hypotension, n (%)	1 (3.3)	0 (0.0)	1.000
Bradycardia, n (%)	2 (6.7)	5 (16.7)	0.421
Hypertension, n (%)	3 (10.0)	3 (10.0)	1.000
Nausea or vomiting, n (%)	4 (13.3)	3 (10.0)	1.000
Neonatal outcomes			
Umbilical artery characteristics			
pH	7.34 ± 0.04	7.34 ± 0.04	0.877
pH < 7.2, n (%)	0 (0.0)	0 (0.0)	1.000
PCO_2_, mmHg	40.86 ± 6.01	43.24 ± 5.71	0.125
PO_2_, mmHg	20.36 ± 4.39	22.33 ± 6.75	0.193
Base excess, mmol/L	−2.48 ± 1.32	−2.45 ± 1.75	0.938
Apgar score			
1 min	9 [9, 9]	9 [9, 9]	0.222
1 min <7, n (%)	1 (3.3)	0 (0.0)	1.000
5 min	10 [10, 10]	10 [10, 10]	0.232
5 min <7, n (%)	0 (0.0)	0 (0.0)	1.000

Values are n (%), mean ± SD, or median [IQR].

## 4 Discussion

This study indicated that the ED90 values for prophylactic norepinephrine and phenylephrine infusions under a higher blood pressure management target were estimated to be 0.140 μg/kg/min (95% CI: 0.133–0.147) and 1.280 μg/kg/min (95% CI: 1.180–1.380), respectively.

Currently, there is a notable lack of evidence regarding the ED90 of prophylactic vasopressors under higher blood pressure management targets during cesarean delivery. Fu et al. (2020) conducted a randomized controlled trial under the condition of maintaining maternal blood pressure above 80% of baseline, comparing norepinephrine infusions of 0.025, 0.05, 0.075, and 0.1 μg/kg/min for preventing maternal hypotension, and without a normal saline control, they determined the ED90 to be 0.080 μg/kg/min (95% CI: 0.065–0.116). Sheng et al. (2024) conducted a randomized controlled trial comparing norepinephrine infusions at doses of 0.02, 0.04, 0.06, 0.08, and 0.10 μg/kg/min for preventing maternal hypotension and, without including a normal saline control group, determined the ED90 to be 0.091 μg/kg/min (95% CI: 0.068–0.147). Furthermore, Sheng et al. (2021) evaluated the efficacy of norepinephrine at doses of 0.02–0.06 μg/kg/min in preventing maternal hypotension during both singleton and twin pregnancies and, without normal saline comparison, determined ED90 values of 0.054 μg/kg/min (95% CI: 0.044–0.079) and 0.058 μg/kg/min (95% CI: 0.046–0.085), respectively.

Under the administration of prophylactic phenylephrine, Xiao et al. (2020) conducted a randomized controlled trial to investigate prophylactic phenylephrine administration at doses of 0.25, 0.375, 0.5, and 0.625 μg/kg/min for maternal hypotension prevention and determined the ED90 to be 0.54 μg/kg/min (95% CI: 0.46–0.76). Allen et al. (2010) compared prophylactic phenylephrine (25, 50, 75, and 100 μg/min) with normal saline for preventing pre-delivery maternal hypotension, demonstrating progressively decreasing incidence rates (80%, 30%, 15%, 11%, and 0%, respectively) with increasing doses, and through Probit regression analysis estimated the ED90 to be 55.95 μg/min (95% CI: 44.48–77.06), equivalent to approximately 0.669 μg/kg/min for an 80 kg maternal weight.

In an up-and-down sequential allocation study, Khatoon et al. (2025) conducted an up-down sequential allocation study comparing norepinephrine (initial dose 1.6 μg/min with 0.2 μg/min increments or decrements) and phenylephrine (initial dose 20 μg/min with 1.5 μg/min increments or decrements) for maternal hypotension prevention, ultimately determining ED50 values of 1.01 μg/min and 12.7 μg/min, respectively. The literature reports the recorded weights of each group as 79.6 kg and 75.9 kg, respectively. The corresponding converted doses were 0.013 μg/kg/min and 0.167 μg/kg/min, respectively. Qian et al. (2022) compared norepinephrine (initial dose 0.1 μg/kg/min with 0.01 μg/kg/min adjustments) and phenylephrine (initial dose 0.5 μg/kg/min with 0.05 μg/kg/min adjustments) for preventing post-anesthesia maternal hypotension following combined spinal-epidural anesthesia, using isotonic regression to determine ED50 values of 0.061 μg/kg/min (95% CI: 0.054–0.068) and 0.368 μg/kg/min (95% CI: 0.343–0.393), respectively. Probit regression analysis further calculated the ED50 values as 0.059 μg/kg/min (95% CI: 0.048–0.068) for norepinephrine and 0.330 μg/kg/min (95% CI: 0.278–0.376) for phenylephrine, and the ED90 values as 0.080 μg/kg/min (95% CI: 0.069–0.120) for norepinephrine and 0.449 μg/kg/min (95% CI: 0.390–0.679) for phenylephrine. The aforementioned ED90 values were uniformly and significantly lower than those reported in this study, likely attributable to the management strategies employed for achieving higher target blood pressures.

During the middle and later stages of pregnancy, physiological adaptations lead to a significant increase in placental blood flow that normally exceeds fetal oxygen requirements. However, in cases of reduced uterine blood flow, such as spinal anesthesia-induced maternal hypotension, fetal compensation through oxygen extraction helps prevent metabolic acidosis (Lee and Ngan Kee, 2019). A transient episode of maternal hypotension, even if the decrease exceeds 30%, does not have an impact the neonatal Apgar score, amniotic fluid contamination, or the occurrence of subsequent oxygen therapy (Maayan-Metzger et al., 2010). Nevertheless, this mechanism may be associated with an increased incidence of maternal adverse effects, including nausea and vomiting. In a sheep model where maternal hypotension is induced, fetal hypoxemia primarily occurs due to maternal hypoxemia. By actively managing hypotension for a short period of time, further fetal stress reactions can be prevented along with decreased PO_2_ levels and increased lactate levels (Erkinaro et al., 2004). Therefore, setting a higher target for blood pressure management is more clinically significant. Proactive treatment can prevent further decreases in maternal blood pressure and reduce the duration of hypotension, thereby ensuring adequate uteroplacental blood flow. This is essential for preventing adverse maternal outcomes and enhancing neonatal outcome.

In this study, the adverse events and neonatal outcomes were comparable between the two groups. Notably, 10% of patients in both groups experienced hypertensive episodes. Owing to the sequential allocation method, some patients received relatively lower doses of vasopressors. However, under the higher blood pressure management target, higher doses of vasopressors were inevitably required, thereby increasing the likelihood of adverse events such as maternal hypertension and bradycardia. Consequently, initiating treatment with a low dose and employing a variable maintenance dose appears to be more effective in preventing hypertensive episodes.

This study has some limitations. First, maternal cardiac output, a critical factor in sustaining uteroplacental perfusion, was not directly monitored. Instead, blood pressure and heart rate were utilized as indirect indicators of cardiac output. Second, the study population included both primiparous women and those with a history of prior cesarean delivery. The inclusion of patients with previous cesarean deliveries may have introduced additional surgical complexity, potentially influencing the dosage calculations for vasopressors. Finally, the incremental or decremental adjustments in the two groups were relatively minor (norepinephrine 0.01 μg/kg/min, phenylephrine 0.1 μg/kg/min), which could have affected the precision of the ED90 estimation and the corresponding 95% confidence interval. The isotonic regression analysis employed in this study utilized the default algorithm without integrating inverse probability weighting, which could potentially result in inaccurate calculation of 95% confidence intervals.

In conclusion, when the target was established to maintain the blood pressure of patients at greater than 90% of the baseline prior to delivery in patients undergoing cesarean delivery, the prophylactic infusion rates for norepinephrine and phenylephrine were determined to be 0.140 and 1.280 μg/kg/min, respectively.

## Data Availability

The original contributions presented in the study are included in the article/supplementary material, further inquiries can be directed to the corresponding author.
